# Impacts of multimorbidity on medication treatment, primary healthcare and hospitalization among middle-aged and older adults in China: evidence from a nationwide longitudinal study

**DOI:** 10.1186/s12889-021-11456-7

**Published:** 2021-07-12

**Authors:** Yang Zhao, Siqi Zhao, Lin Zhang, Tilahun Nigatu Haregu, Haipeng Wang

**Affiliations:** 1 The George Institute for Global Health at Peking University Health Science Center, Beijing, China; 2 The Nossal Institute for Global Health, The University of Melbourne, Melbourne, VIC Australia; 3 WHO Collaborating Centre on Implementation Research for Prevention and Control of Noncommunicable Diseases, Melbourne, VIC Australia; 4 Yantaishan Hospital of Yantai, Yantai, Shandong China; 5 Yantai Sino-French Friendship Hospital, Yantai, Shandong China; 6 Peking Union Medical College School of Public Health, Chinese Academy of Medical Sciences and Peking Union Medical College, Beijing, China; 7 Centre for Epidemiology and Biostatistics, Melbourne School of Population and Global Health, The University of Melbourne, Melbourne, VIC Australia; 8 Centre for Health Management and Policy Research, School of Public Health, Cheeloo College of Medicine, Shandong University, Jinan, 250012 China; 9 NHC Key Lab of Health Economics and Policy Research (Shandong University), Jinan, 250012 Shandong China

**Keywords:** Multimorbidity, Primary care, Health expenditure, Longitudinal analysis

## Abstract

**Background:**

Multimorbidity is a significant contributor to inequalities in healthcare and has become a major unaddressed challenge for the health system in China. The aim of this study is to assess the socio-demographic distribution of multimorbidity and the relationships between multimorbidity, primary healthcare, hospitalization and healthcare spending.

**Methods:**

We conducted this nationwide population-based panel data study in China. Study participants included 12,306 residents aged ≥45 years from the China Health and Retirement Longitudinal Study in 2011, 2013 and 2015. Random-effects logistic regression models were applied to estimate the association between multimorbidity and primary healthcare as well as admission to the hospital. We used log-linear regression models to investigate the association between multimorbidity and health spending.

**Results:**

Overall, 46.2% of total interviewees reported multimorbidity. Random-effects logistic regression analyses showed that multimorbidity was associated with a higher likelihood of medication use (Adjusted odds ratio (AOR) =19.19, 95% CI = 17.60, 20.93), health check (AOR = 1.51, 95% CI = 1.43, 1.59), outpatient care (AOR = 2.39, 95% CI = 2.23, 2.56) and admission to hospital (AOR = 2.94, 95% CI = 2.68, 3.21). Log-linear regression models showed that multimorbidity was also positively associated with spending for outpatient care (coefficient = 0.64, 95% CI = 0.59, 0.68) and hospitalization (coefficient = 0.65, 95% CI = 0.60, 0.71).

**Conclusions:**

Multimorbidity is associated with higher levels of primary care, hospitalization and greater financial burden to individuals in China. Health systems need to shift from single-disease models to new financing and service delivery models to more effectively manage multimorbidity.

**Supplementary Information:**

The online version contains supplementary material available at 10.1186/s12889-021-11456-7.

## Introduction

Chronic conditions are both a major contributor to inequalities in healthcare and the leading cause of premature mortality in China [1, 2]. The ageing of the population increases exposure to risk factors, so the prevalence of multimorbidity, defined as two or more co-existing chronic conditions, is likely to increase rapidly over the coming decades [3]. Despite the growing prevalence of multimorbidity in China, there is little evidence regarding the impact of multimorbidity on access to and inequalities in primary care and health service use compared to the impact of a single chronic condition.

Under the sustainable development goals (SDG) agenda, advancing universal health coverage (UHC) is the centrepiece of health policy in many countries [4]. To improve access to primary care and equity in financial protection, the China New Health System Reform resulted in a significant increase in health insurance coverage, with 95.7% of the Chinese population being covered by one of the three main health insurance schemes in 2011 (that is, the Urban Employee Basic Medical Insurance (UEBMI) scheme, the Urban Resident Basic Medical Insurance (URBMI) scheme, and the New Rural Cooperative Medical Scheme (NCMS)) [5, 6]. However, the benefits package and degree of financial protection for patients with chronic non-communicable diseases (NCDs) varies between these schemes [7, 8]. People enrolled in the URBMI and NCMS have a lower level of financial protection than those individuals with UEBMI as a result of annual reimbursement limits and an incomplete list of services and drugs covered by the schemes, and so on.

While there have been many studies conducted in high-income countries (HICs) on the associations between NCD multimorbidity, healthcare utilisation, and financial protection [9–11], this topic is still an emerging area of research inquiry in low-to-middle income countries (LMICs). Currently, only a few small studies in certain parts of China have examined this issue. For example, a study conducted in Guangdong investigated health care utilisation arising from multimorbidity of 162,464 subjects [12]. Very few studies have estimated the impact of multimorbidity on primary healthcare and Out-of-Pocket Expenditure (OOPE) at the national level [13, 14]. To our knowledge, this is the first panel analysis to examine the association between multimorbidity and primary healthcare, hospitalisation as well as health spending, using nationally representative longitudinal data in China.

## Methods

### Study design and participants

The data from three waves of the China Health and Retirement Longitudinal Study (CHARLS) 2011–2015 were used. CHARLS collected high-quality data, from a nationally representative sample of Chinese residents aged 45 years and older, using multi-stage stratified probability-proportionate-to-size sampling [15]. Total sample size was 17,708 individual (overall response rate: 80.5%) at the baseline survey of CHARLS in 2011. The follow-up surveys were conducted every 2 or 3 years. The data also included individual weighting variables to ensure that it was nationally representative. A detailed description of the CHARLS methods was reported elsewhere [15]. The CHARLS questionnaires included the information related to participant’s’ socioeconomic and health status, health service utilisations, medical spending and insurance, and several biomarkers, such as blood pressures. The Biomedical Ethics Review Committee of Peking University approved CHARLS (approval number: IRB00001052–11015), and all participants were required to provide written informed consent. In our study, there were 13,565 individuals who reported no losses to follow-up over the three waves of CHARLS. After removing those participants with missing values in independent and/or dependent variables, the final sample included 12,306 respondents (90.7% of respondents without any loss to follow-up).

### Variables

In this study, multimorbidity was defined as the coexistence of two or more chronic non-communicable diseases (NCDs) in one patient [16–18]. A total of 11 NCDs were used to measure multimorbidity, including diagnosed hypertension and 10 self-reported diagnosed chronic diseases (diabetes, dyslipidaemia, cancer, stroke, heart disease, liver disease, kidney disease, chronic lung disease, digestive disease, and arthritis). This study did not include individuals with self-reported psychiatric and memory-related diseases due to potential recall bias. We counted the number of NCDs for each individual to identify those with multimorbidity. In CHARLS, the trained nurses recorded participants’ systolic blood pressure and diastolic blood pressure three times using a HEM-7112 electronic monitor (OMRON, Tokyo, Japan). Diagnosed hypertension was defined as systolic blood pressure ≥ 140 mmHg and/or diastolic blood pressure ≥ 90 mmHg, and/or being on antihypertensive medication for raised blood pressure [19, 20].

Participants were asked about their health services use: 1) number of outpatient visits in the past month, 2) any hospital stay in the past year, and 3) any health check in the past 2 years. Apart from health service use, participants were also asked about any medication use for a specific chronic disease or its complications when the survey was conducted. Furthermore, CHARLS also collected information on how much respondents paid totally and out-of-pocket for their outpatient visit during the last month and hospitalization during the last year. Healthcare expenditures and out-of-pocket payments, after reimbursement from insurance, were categorized by these two types of healthcare utilization.

The following variables as covariates were included: age, gender, marital status (married and partnered, unmarried and others), education level (primary school and below, secondary school, college and above), residence location (rural, urban), socio-economic status quartiles (yearly per capita household consumption expenditure), health insurance (UEBMI, URBMI, NCMS, other insurance, without insurance), and geographical region. Five classes within the geographical region were identified and ranked, based on their Gross Domestic Product (GDP) per capita at the province level in China: Class 1, > 12,000 US$; Class 2, 12,000–10,000 US$; Class 3, 10,000–7000 US$; Class 4, 7000–6000 US$; and Class 5, < 6000 US$ [21].

### Statistical analyses

The Chi-square test was applied to estimate the socioeconomic difference in multimorbidity prevalence, primary care and hospitalisation across NCD groups. The random-effects logistic regression model was used to examine relationships between multimorbidity and primary healthcare and admission to the hospital. We used log-linear regression models to investigate the relationship between multimorbidity and healthcare spending. Descriptive analysis of socio-demographic characteristics and prevalence of multimorbidity was weighted to account for the multi-stage PPS design of CHARLS and loss of follow-up. All statistical analyses were performed using STATA 15.0 (Stata Corp 2017) and statistically significance was considered as *P* ≤ 0 .05.

## Results

Table 1 displays characteristics of the investigated population and the prevalence of multimorbidity among middle-aged and elder people in China. As many as 46.2% of total interviewees reported multimorbidity, 39.3% for those aged 45–59, and 55.6% for those aged ≥60, indicating that multimorbidity had become a dangerous threat to over the half of ageing population and got more severe with the ageing process. Among the multimorbidity group, the majority reported 2 (30.2%) and 3 conditions (23.4%), compared to those with 4 and more conditions (12.4%). The prevalence of multimorbidity was significantly higher among individuals who were female and unmarried, lower educated and insured by URBMI or UEBMI. A significant difference was also found across regions with different economic development. We compared those participants with and without missing data in terms of key socio-demographic variables and the results showed no significant difference of age (for 45–65 years, 57.7% versus 57.8%; for ≥60 years, 42.3% versus 42.2%) and gender (53.7% versus 51.6%) between both groups. There were statistical differences for some other socio-demographic variables, such as residence location, marital and education status, which we also considered and included these covariates when conducting the multivariable regression analyses (Table S1).

**Table 1:** Characteristics of the baseline survey from the CHARLS (*n* = 12,306)

Characteristics	All participants (N, %)		Percentage of multimorbidity (95% CI)			***P*** value
Age, years						
45–59	7125	57.8	39.3	37.7	41.0	< 0.001
≥ 60	5181	42.2	55.6	53.8	57.4	
Gender						
Male	5972	48.4	43.9	42.0	45.8	< 0.001
Female	6334	51.6	48.4	46.7	50.1	
Marital status						
Married and partnered	10,916	87.6	45.0	43.6	46.3	< 0.001
Unmarried and others	1390	12.4	54.8	51.6	57.9	
Education level						
Primary school and below	8406	66.6	48.9	47.4	50.3	< 0.001
Secondary school	2549	20.9	40.5	37.9	43.1	
College and above	1351	12.5	41.5	37.1	46.0	
Residence status						
Urban	4338	42.5	47.0	44.5	49.4	0.369
Rural	7968	57.5	45.6	44.5	46.8	
Region						
Class 1 (the most affluent)	1243	12.0	43.4	39.7	47.1	< 0.001
Class 2	2670	22.8	40.7	37.0	44.6	
Class 3	1613	13.3	48.6	45.7	51.5	
Class 4	5035	37.6	49.1	47.5	50.6	
Class 5 (the most deprived)	1745	14.3	47.5	44.9	50.0	
PCE, quintile						
Q1 (the lowest)	2968	23.2	43.7	41.5	46.0	0.348
Q2	2969	22.9	46.1	44.1	48.1	
Q3	2966	24.9	45.4	42.8	48.0	
Q4 (the highest)	2967	29.0	48.8	46.1	51.5	
Health insurance						
None	729	6.2	44.2	39.0	49.6	< 0.001
UEBMI	1027	11.8	52.5	47.6	57.3	
URBMI	589	5.9	54.5	48.3	60.6	
NCMS	9653	73.2	44.5	43.2	45.8	
Others	308	2.8	50.8	43.4	58.2	
Number of NCDs						
0	2850	23.6				
1	3759	30.2				
2	2880	23.4				
3	1548	12.4				
4	773	6.3				
5	322	2.5				
≥ 6	174	1.6				

a, Values are unweighted counts and weighted percentages unless otherwise indicated; *CHARLS* China Health and Retirement Longitudinal Study, *PCE* Per capita household annual consumption expenditure, *UEBMI* Urban Employee Basic Medical Insurance, *URBMI* Urban Resident Basic Medical Insurance, *NCMS* New Rural Cooperative Medical Scheme; Others, government healthcare, private medical insurance and so on

**Fig. 1:**
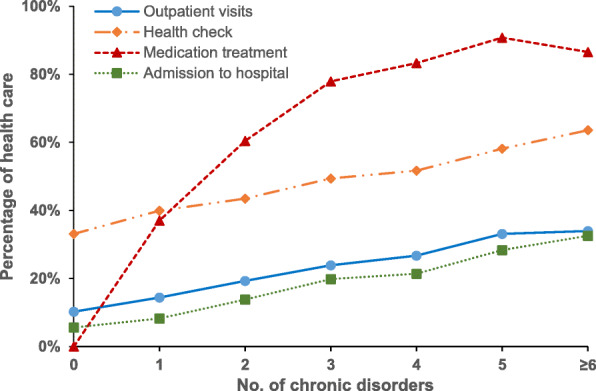
Percentage of primary care use and hospitalization by the number of chronic diseases, 2015

**Fig. 2:**
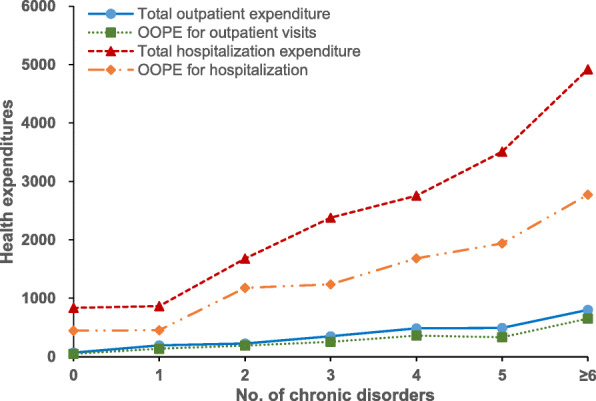
Total expenditure and OOPE for outpatient visits and hospitalization by the number of chronic diseases, 2015

Figure 1 showed that the proportion of outpatient visits, health check and hospitalization increased substantially for persons with increasing numbers of co-existing chronic disorders, which indicated a positive association between number of co-morbidity and healthcare utilization. The percentage of medication use increased remarkably with an increasing number of chronic diseases (≥5 disorders), while it decreased slightly when the number of co-morbidities was over 6. For the health expenditure, Fig. 2 revealed that there were increasing trends in both total expenditure and OOPE for healthcare with an increasing number of chronic diseases in China in 2015. Compared with spending on outpatient visits, the payment for inpatient care had a significantly higher degree of total expenditure and OOPE.

After controlling for demographic, socioeconomic and health insurance factors, multimorbidity had a positive relationship with health service utilization. For instance, the health check-ins, outpatient visits and admissions to hospital among Chinese adults with multimorbidity were 1.51-fold, 2.39-fold and 2.94-fold higher, respectively, than those for people with no multimorbidity. Compared to those without multimorbidity, persons with multimorbidity were more likely to take medication (AOR = 19.19, 95% CI =17.60, 20.93). Rural residents were more likely to use outpatient care (AOR = 1.10, 95% CI =1.01, 1.19) but have less health check-ins (AOR = 0.80, 95% CI =0.75, 0.86) and be hospitalized (AOR = 0.88, 95% CI =0.79, 0.97) than people living in urban areas. The older age (60 and over), female gender, high household economic status, having health insurance were positively associated with health check-ins and medication use. The rural-urban disparity was found as well from regression models (Table 2).

**Table 2:** Association of multimorbidity with primary care and hospitalisation in China

Variables (reference)	Health check			Medication treatment			Outpatient care			Admission to hospital		
	AOR	95% CI		AOR	95% CI		AOR	95% CI		AOR	95% CI	
Multimorbidity (none)	1.51***	1.43	1.59	19.19***	17.60	20.93	2.39***	2.23	2.56	2.94***	2.68	3.21
Age (45–59 years)	1.51***	1.42	1.60	1.46***	1.34	1.59	1.04	0.96	1.11	1.62***	1.48	1.78
Gender (male)	1.15***	1.09	1.22	1.39***	1.27	1.52	1.31***	1.22	1.40	0.94	0.86	1.03
Marital status (married)	0.99	0.91	1.08	0.97	0.86	1.10	1.07	0.96	1.18	1.14	1.00	1.29
Education (primary school and below)												
Secondary school	1.13**	1.05	1.22	0.86*	0.77	0.97	0.93	0.85	1.02	0.87*	0.77	0.98
College and above	1.48***	1.33	1.64	0.75***	0.64	0.88	0.96	0.85	1.09	0.77**	0.65	0.90
Residence place (urban)	0.80***	0.75	0.86	1.03	0.93	1.14	1.10*	1.01	1.19	0.88*	0.79	0.97
Region (Class 1, the most affluent)												
Class 2	0.40***	0.36	0.45	1.09	0.92	1.29	1.15*	1.00	1.31	1.26*	1.06	1.51
Class 3	0.39***	0.35	0.44	1.06	0.89	1.28	1.00	0.86	1.16	1.73***	1.43	2.08
Class 4	0.45***	0.40	0.49	1.15	0.99	1.34	1.36***	1.20	1.54	1.66***	1.41	1.96
Class 5 ((the most deprived)	0.37***	0.33	0.42	1.33**	1.11	1.59	1.00	0.86	1.15	1.83***	1.51	2.20
PCE, quartile (Q1, the lowest)												
Q2	1.08*	1.01	1.16	1.13*	1.02	1.24	1.16**	1.06	1.26	1.18**	1.05	1.33
Q3	1.16***	1.08	1.25	1.17**	1.06	1.29	1.12*	1.02	1.22	1.59***	1.41	1.78
Q4 (the highest)	1.30***	1.21	1.41	1.22***	1.10	1.36	1.26***	1.14	1.38	1.98***	1.76	2.23
Health insurance(none)												
UEBMI	2.35***	2.03	2.73	1.43***	1.17	1.76	1.29**	1.07	1.56	1.75	1.39***	2.20
URBMI	1.35***	1.15	1.58	1.37**	1.11	1.69	1.22	0.99	1.49	1.40	1.09**	1.79
NCMS	1.36***	1.21	1.52	1.22**	1.05	1.42	1.51***	1.31	1.75	1.50	1.24***	1.81
Others	2.16***	1.76	2.67	1.51**	1.14	2.01	1.31**	1.01	1.70	1.35	0.98	1.86

*PCE* Per capita household annual consumption expenditure, *UEBMI* Urban Employee Basic Medical Insurance, *URBMI* Urban Resident Basic Medical Insurance, *NCMS* New Rural Cooperative Medical Scheme; Others, government healthcare, private medical insurance and so on. ***P < 0.001, **P < 0.01, *P < 0.05 significance test

The log-linear regression models showed that chronic disease multimorbidity was positively associated with a higher total and out-of-pocket payment for both outpatient and inpatient care, after controlling for socioeconomic factors. Compared to patients with non-multimorbidity, individuals with multimorbidity had more OOPE for outpatient visits (coefficient = 0.64, 95% CI = 0.59, 0.68) and hospitalization (coefficient = 0.65, 95% CI = 0.60, 0.71) (Table 3).

**Table 3:** The association between multimorbidity and health spending in China

Variables (reference)	Total outpatient spending			OOPE for outpatient care			Total inpatient spending			OOPE for inpatient care		
	Coefficient	95% CI		Coefficient	95% CI		Coefficient	95% CI		Coefficient	95% CI	
Multimorbidity (none)	0.67***	0.62	0.72	0.64***	0.59	0.68	0.73***	0.67	0.79	0.65***	0.60	0.71
Age (45–59 years)	0.05	0.00	0.10	0.03	−0.02	0.09	0.34***	0.28	0.41	0.28	0.22***	0.34
Gender (male)	0.20***	0.14	0.25	0.20***	0.15	0.25	−0.07*	− 0.14	− 0.01	− 0.05	− 0.11	0.01
Marital status (married)	− 0.01	− 0.09	0.07	− 0.04	− 0.11	0.04	0.04	− 0.06	0.14	− 0.03	− 0.12	0.06
Education (primary school and below)												
Secondary school	−0.01	− 0.08	0.06	− 0.01	− 0.08	0.05	− 0.05	−0.14	0.03	−0.05	− 0.13	0.02
College and above	0.02	−0.08	0.11	0.00	−0.09	0.09	−0.16**	−0.28	− 0.04	−0.16**	− 0.27	−0.05
Residence place (urban)	0.09**	0.03	0.15	0.08**	0.03	0.14	−0.07	−0.14	0.01	−0.05	− 0.12	0.02
Region (Class 1, the most affluent)												
Class 2	0.08	−0.02	0.18	0.11*	0.01	0.20	0.18**	0.06	0.31	0.17**	0.06	0.28
Class 3	−0.01	−0.12	0.09	0.02	−0.09	0.12	0.33***	0.19	0.46	0.29***	0.17	0.41
Class 4	0.17***	0.08	0.27	0.20***	0.11	0.28	0.32***	0.21	0.43	0.28***	0.17	0.38
Class 5 ((the most deprived)	−0.02	−0.13	0.08	0.00	− 0.10	0.10	0.35***	0.22	0.49	0.33***	0.20	0.45
PCE, quartile (Q1, the lowest)												
Q2	0.10**	0.03	0.17	0.10**	0.03	0.16	0.12**	0.04	0.20	0.11**	0.04	0.19
Q3	0.14***	0.07	0.21	0.14***	0.08	0.21	0.37***	0.29	0.45	0.35***	0.27	0.42
Q4 (the highest)	0.27***	0.20	0.34	0.26***	0.19	0.33	0.61***	0.52	0.69	0.57***	0.49	0.64
Health insurance(none)												
UEBMI	0.22**	0.09	0.35	0.07	−0.06	0.20	0.48***	0.31	0.64	0.40***	0.25	0.55
URBMI	0.13	−0.01	0.28	0.11	−0.02	0.25	0.22*	0.04	0.39	0.22***	0.06	0.38
NCMS	0.26***	0.16	0.36	0.25***	0.15	0.35	0.26***	0.14	0.38	0.22***	0.11	0.34
Others	0.15	−0.04	0.34	−0.01	−0.20	0.17	0.27*	0.04	0.50	0.12	−0.09	0.33

*PCE* Per capita household annual consumption expenditure, *UEBMI* Urban Employee Basic Medical Insurance, *URBMI* Urban Resident Basic Medical Insurance, *NCMS* New Rural Cooperative Medical Scheme; Others, government healthcare, private medical insurance and so on; ***P < 0;001, **P < 0;01, *P < 0;05 significance test

## Discussion

Using nationally representative data, we found that the burden of chronic disease multimorbidity was significantly high among middle-aged and elderly Chinese adults. Multimorbidity was common, particularly among the senior elderly population group. We identified positive associations between multimorbidity and healthcare utilization. The number of chronic diseases experienced by a person was associated with more use of primary healthcare and hospitalization service, and greater expenditures for both outpatient care and inpatient care.

The study showed that nearly half of persons aged ≥45 years experienced multimorbidity in China. Multimorbidity was positively associated with age, female gender, unmarried status, low level of education and health insurance. Our findings on socioeconomic differences in multimorbidity prevalence are consistent with other province-level studies in China [12]. The effect of increasing age on the prevalence of multimorbidity could be explained by the fact that there is an accumulation of chronic conditions during the ageing process [22]. Several studies from different countries have shown that individuals who are married tend to have a lower prevalence of chronic disease and all-cause mortality than those who are not married [23, 24]. Being married is associated with having health-protective effects. These include economic benefits [25], social support, and social control by a spouse [24, 26]. However, the associations between education and multimorbidity often vary across LMICs [22, 27]. To our knowledge, there is a dearth in the literature on the relationship between economic development in the region and multimorbidity prevalence in China.

Previous research conducted across the world has revealed substantial disparities in the prevalence of multimorbidity due to several factors including inconsistent definitions, different measurement methods, the sampling frame, sources of patient data, and study setting [12, 16, 22]. It is found that the rate of multimorbidity was 37% among older people in European countries [28]. A systematic review shows that the prevalence of multimorbidity in cross-sectional studies is around 20–30% when the whole population is taken into account [22]. Therefore, the true variation in clusters of chronic conditions is worth considering, which needs more research in the future.

Earlier studies have demonstrated that an increasing number of chronic diseases is associated with higher healthcare utilisation, and this association has been well-documented in HICs [29–31]. Our finding revealed positive relationships between multimorbidity and outpatient care as well as hospitalization, which are consistent with previous local studies in China [11–14]. This study provides new evidence on the association between multimorbidity and health check and medication treatment for chronic conditions. Consistent with earlier published studies, the presence of multimorbidity was associated with higher levels of health expenditures [12, 29, 31]. This is also likely to be due to patients with multimorbidity requiring more prescription medications and having higher drug expenditures compared to those with just one or no chronic conditions [10, 32]. Multimorbidity impairs quality of life and functional ability, leads to frailty and escalates healthcare costs [12, 33]. However, the percentage of medication use decreased slightly when the patients have 6 and more co-existing chronic diseases.

This study examined the impact of multimorbidity on primary care, hospitalization and health expenditure using a nationwide longitudinal sample of middle-aged and elderly Chinese adults. However, there are several limitations to acknowledge in the study. First, the data were collected using a structured questionnaire based on self-reported information of the participants, which might be subjected to recall bias. Second, the questionnaire did not include all chronic diseases in clinical studies, which might underestimate the prevalence of multimorbidity. Third, healthcare utilization (except medication use) and health expenditure were not specific for chronic diseases, which might exaggerate the impact of multimorbidity on them. Fourth, we examined the effect of multimorbidity on healthcare utilisation and expenditure by simply counting the number of chronic diseases, but without accounting for the type and severity of chronic diseases.

This study provides further evidence of the need for relevant policies and targeted interventions to tackle the growing burden of multimorbidity. Despite the increasing prevalence of multimorbidity, current health policy and clinical practice still largely emphasize a single-disease specific approach [11, 32]. Disease-specific guidelines are usually not appropriate for the management of individuals with multimorbidity, so several countries have developed clinical guidelines for multimorbidity which emphasize the integration of healthcare delivery [34]. Therefore, Chinese health system need to shift from single-disease models to new financing and service delivery models for effectively managing multimorbidity [35]. Improving the continuity and coordination of care for people with multimorbidity is a key challenge for the healthcare system worldwide, and each patient needs a dedicated clinician to take responsibility for care coordination [16]. Therefore, a strong primary care system led by a mix-skilled healthcare professional team is essential for delivering integrated care for people with multimorbidity [11, 12, 36].

Multimorbidity is costly to health systems and individuals. Out-of-pocket health expenditure on medicines can severely compromise financial risk protection [8, 37]. Patients suffering from multimorbidity may have greater health expenditure burden due to their complex treatment needs. Better continuity of care for those with chronic diseases may ultimately lead to lower episode-based costs [12]. Targeted government funding and support programmes should take into account financial protection for patients with multimorbidity, particularly for the elderly and low socioeconomic status groups. Health insurance must be designed to provide enhanced and broadened coverage for multimorbidity to promote fair financing and better access to health services [11, 32]. Further research is required to better understand the cost-effectiveness of different strategies to reduce the burden of multimorbidity on individuals and health systems.

In conclusion, multimorbidity is a major unaddressed challenge to individuals and health systems in China and other LMICs. Health systems need to shift from single-disease models to new financing and service delivery models to more effectively manage multimorbidity. Healthcare reforms in China should place greater emphasis on strengthening primary care, optimizing patient-centeredness in integrated healthcare delivery and improving health insurance coverage for people with multiple chronic diseases. Multimorbidity patients with low socioeconomic status deserve more attention from health policymakers, providers and educators of health professionals in China and other LMICs.

## Supplementary Information

**Additional file 1: Table S1.** Characteristics of the baseline sample included and excluded due to losses to follow-up.

## Data Availability

The datasets used and/or analysed during the current study are available from the corresponding author on reasonable request.

## Abbreviations

SDG: Sustainable development goals
UHC: Universal health coverage
NRCMS: New Rural Cooperative Medical Scheme
URBMI: Urban Resident Basic Medical Insurance
UEBMI: Urban Employee Basic Medical Insurance
AOR: Adjusted odds ratio
CHARLS: China Health and Retirement Longitudinal Study
NCDs: Non-communicable diseases
HICs: High-income countries
LMICs: Low-to-middle income countries
OOPE: Out-of-pocket expenditure
PSUs: Primary sampling units
