# Supplementary material for: Impacts of multimorbidity on medication treatment, primary healthcare and hospitalization among middle-aged and older adults in China: evidence from a nationwide longitudinal study
Source: BMC Public Health. 2021 Jul 12;21:1380. doi: 10.1186/s12889-021-11456-7 (PMC8274017; doi:10.1186/s12889-021-11456-7)
Supplement: Supplementary file 1 — Additional file 1: Table S1. Characteristics of the baseline sample included and excluded due to losses to follow-up. [file 12889_2021_11456_MOESM1_ESM.docx]

**Table S1** Characteristics of the baseline sample included and excluded due to losses to follow-up

| **Characteristics** | **Without losses to follow-up**  **(N, %)** | |  | **With losses to follow-up**  **(N, %)** | | **P value** |
| --- | --- | --- | --- | --- | --- | --- |
| Age, years |  |  |  |  |  |  |
| 45-59 | 7,125 | 57.8 |  | 3,105 | 57.7 | 0.840 |
| ≥60 | 5,181 | 42.2 |  | 2,273 | 42.3 |  |
| Gender |  |  |  |  |  |  |
| Male | 5,972 | 48.4 |  | 2,504 | 46.4 | 0.008 |
| Female | 6,334 | 51.6 |  | 2,898 | 53.7 |  |
| Marital status |  |  |  |  |  |  |
| Married and partnered | 10,916 | 87.6 |  | 4,532 | 84.1 | <0.001 |
| Unmarried and others | 1,390 | 12.4 |  | 858 | 15.9 |  |
| Education level |  |  |  |  |  |  |
| Primary school and below | 8,406 | 66.6 |  | 3,365 | 62.3 | <0.001 |
| Secondary school | 2,549 | 20.9 |  | 1,128 | 20.9 |  |
| College and above | 1,351 | 12.5 |  | 909 | 16.8 |  |
| Residence status |  |  |  |  |  |  |
| Urban | 4,338 | 42.5 |  | 2,833 | 52.4 | <0.001 |
| Rural | 7,968 | 57.5 |  | 2,569 | 47.6 |  |
| Region |  |  |  |  |  |  |
| Class 1 (the most affluent) | 1,243 | 12.0 |  | 655 | 12.1 | 0.001 |
| Class 2 | 2,670 | 22.8 |  | 1,150 | 21.3 |  |
| Class 3 | 1,613 | 13.3 |  | 724 | 13.4 |  |
| Class 4 | 5,035 | 37.6 |  | 2,116 | 39.2 |  |
| Class 5 (the most deprived) | 1,745 | 14.3 |  | 757 | 14.0 |  |
| PCE, quintile |  |  |  |  |  |  |
| Q1 (the lowest) | 2,968 | 23.2 |  | 986 | 20.9 | <0.001 |
| Q2 | 2,969 | 22.9 |  | 1,087 | 23.0 |  |
| Q3 | 2,966 | 24.9 |  | 1,185 | 25.1 |  |
| Q4 (the highest) | 2,967 | 29.0 |  | 1,472 | 31.1 |  |
| Health insurance |  |  |  |  |  |  |
| None | 729 | 6.2 |  | 450 | 8.7 | <0.001 |
| UEBMI | 1,027 | 11.8 |  | 857 | 16.6 |  |
| URBMI | 589 | 5.9 |  | 393 | 7.6 |  |
| NCMS | 9,653 | 73.2 |  | 3,249 | 63.0 |  |
| Others | 308 | 2.8 |  | 212 | 4.1 |  |
| Number of NCDs |  |  |  |  |  |  |
| None | 2,850 | 23.2 |  | 662 | 18.4 | <0.001 |
| Single disease | 3,759 | 30.6 |  | 999 | 27.7 |  |
| multimorbidity | 5,697 | 46.3 |  | 1,946 | 54.0 |  |

a, Values are unweighted counts and unweighted proportions; UEBMI, Urban Employee Basic Medical Insurance; URBMI, Urban Resident Basic Medical Insurance; NCMS, New Rural Cooperative Medical Scheme; Others, government healthcare, private medical insurance and so on.
